# Assessing Vaccine Herd Protection by Killed Whole-Cell Oral Cholera Vaccines Using Different Study Designs

**DOI:** 10.3389/fpubh.2019.00211

**Published:** 2019-07-31

**Authors:** Mohammad Ali, John Clemens

**Affiliations:** ^1^Johns Hopkins Bloomberg School of Public Health, Baltimore, MD, United States; ^2^International Centre for Diarrhoeal Disease Research, Dhaka, Bangladesh

**Keywords:** herd effects, clinical trial, gis, cholera, vaccine

## Abstract

The population level effectiveness of a vaccine may arise as the result of direct protection of vaccinees and vaccine herd protection, which may protect non-vaccinees, vaccinees, or both. Indirect, total, enhanced, and overall vaccine protection are measures of vaccine herd protection. The level of population level effectiveness induced by a vaccine is driven by several factors, including known vaccine-induced protective efficacy, the magnitude, and distribution of vaccine coverage at a point in time and the extent to which different groups mix with one another in the community. Data on vaccine herd protection are valuable in understanding the importance and cost-effectiveness in deploying the e vaccine in public health program. Killed whole-cell (WC) oral cholera vaccines (OCVs) have been evaluated for herd protection in various study settings, leveraging geographic information system (GIS) tools for the analyses. This article provides a brief description of the herd protective effects of killed WC OCVs measured using various study deigns that include (a) individually randomized, controlled clinical trials, (b) cluster randomized clinical trials, (c) observational cohort studies, and (d) observational case-control studies. In all of the study designs, significant herd protection was observed in unvaccinated persons as well as in the community as a whole. The findings of these studies suggest that using killed WC OCV as a public health tool for controlling cholera is impactful and cost-effective.

## Introduction

For pathogens transmitted from person to person, vaccination of a community may reduce pathogen transmission and thereby confer protection to the community beyond that expected on the basis of direct vaccine protection of vaccinees. This phenomenon is known as herd protection. The level of herd protection induced by a vaccine depends on several factors, including known vaccine protective efficacy, the level and distribution of vaccine coverage in the community, and the degree to which different groups in the community mix with each other (1). Assessing herd protection of a vaccine has increased due to the cost of vaccines, especially in resource constrained settings, as well as for vaccines with moderate levels of efficacy for which additional herd effects may need to be considered to make a strong public health case for vaccine introduction. For the oral cholera vaccines (OCVs) that confer only a moderate level of protection are a good example (2). Although killed WC OCVs had been licensed since the 1990s, their herd protective effects were not appreciated until 2005. At that time we demonstrated such herd effects in a clinical study of killed WC OCVs that we had conducted 20 years earlier in rural Bangladesh (3). Since killed WC OCVs offered moderate level of protection (~50%) that persisted for 2 to 3 years in the 1985 trial (4), the vaccines were not introduced as a tool to control cholera in cholera affected countries, with the exception of Vietnam. Had the additional benefit of vaccine herd protection been known at that time, these OCVs might have been included into the immunization programs in countries with cholera. Nowadays, the killed WC OCVs are widely used in cholera affected countries for controlling cholera outbreaks and endemic cholera, an effort that is benefitted by the global killed WC OCV stockpile (5).

## Measures of Herd Protection by Killed WC OCV

A vaccine efficacy study is traditionally designed to evaluate “protective efficacy,” a measure that reflects direct protection of vaccinees in isolation from other vaccinees (6). Since cholera is transmitted from a person to another person, either direct or indirect way, the occurrence of cholera is dependent on the size of population already infected (7). Because of this, vaccines may protect persons directly as well indirectly to people living in the community receiving vaccine due to reduction of magnitude of transmission of the infection among members in the community (8, 9). These vaccine herd protection effects can be thought of as resulting from the following: (a) vaccination likely reduced the number of susceptible population in the vaccinated communities, therefore reducing the possibility that an infectious case would come into contact with a susceptible person; and (b) vaccination likely reduces the prevalence of infectious cases in the community at any given point of time, further reducing the possibility that an infectious individual would come into contact with a susceptible individual. From the public health perspective it is critical to know both direct and indirect protective effectiveness of vaccines, which together determine the population level protective effectiveness of the vaccine.

Vaccine herd protection can be manifested as protection of non-vaccinees (termed *indirect vaccine protection*), or enhanced protection of vaccinees, owing to their reduced level of exposure to the pathogen targeted by the vaccine in a population in which a sufficient level of vaccination has been achieved (10–14). Direct vaccine protection plus the *enhanced protection* of vaccinees owing to reduced transmission is termed *total vaccine protection*. *Overall vaccine protection* is the protection offered by a vaccine in a vaccinated community irrespective of individuals' vaccination status owing to both direct and herd vaccine effects. Indirect, total and overall effects are measures of population-level protective effects of vaccines (15). How vaccine herd protection conferred by killed WC OCVs has been measured in various study designs is described in this article.

## Virtual Cluster Level Coverage (VCLC)

Before describing how population level vaccine effectiveness is measured under various study design, it is important to review the concept of virtual cluster level coverage (VCLC), a major consideration for the analysis of vaccine herd protection in many of the designs. In assessing the herd protection by killed WC OCVs, it was necessary to calculate the actual coverage with OCV of individuals residing in the immediate vicinity around each analyzed individual, where GIS is used to define the coordinates of the perimeter of the virtual cluster. We reasoned that if herd protection in killed WC OCVs was operative, analyzed individuals surrounded by virtual clusters with higher vaccine coverage should have a lower risk of cholera. We further reasoned that, ideally, the virtual cluster sizes should correspond to the “geographic dimensions of chains of person-to-person transmission of cholera affecting the analyzed individuals, because OCV herd protection results from interruption of person-to-person transmission” (16). However, these dimensions likely vary considerably from setting to setting and are rarely known, so that in the studies outlined in this article we used a different statistical criterion to come up with the area around each analyzed individual to define virtual cluster of people for computing the VCLC of the killed WC OCV (17). The approach is related to the difference in the variances of vaccine coverage across clusters, this it does not depend on the relationship between vaccine coverage and the risk of cholera. The approach yielded a 500 m radius for a study in Matlab, Bangladesh, 250 m for a study in Kolkata, India, and 400 m for a study in Zanzibar, East Africa as the virtual cluster around each analyzed individual in order for evaluating herd protective effects of killed WC OCV under evaluation.

Recently, we conducted a study using killed WC OCV as “vaccine probe” to determine geographic boundaries of person-to-person transmission of cholera in two different settings in which placebo-controlled, randomized trials of killed WC OCV had been conducted: Matlab, Bangladesh (which tested a killed cholera toxin B subunit (BS)-killed WC OCV and a killed WC-only OCV) and Kolkata, India (which tested a killed WC OCV) (16). In the study, the virtual clusters were defined as groups (rings) of people around individuals who were analyzed for the risk of cholera. Centering on the residential location of each placebo recipient, the rings were formed starting with 100 meters increments up to 700 meters in Matlab, Bangladesh, and starting with 50 meters increments up to 350 meters in Kolkata, India. The difference of the ring sizes between the two study areas was related to the difference in spatial distribution of households, i.e., dispersedly located households in the rural Matlab vs. the densely populated urban setting in Kolkata. OCV coverage in virtual cluster was then computed as the number of two-dose recipients of OCV divided by the number of all age-eligible individuals for vaccine or placebo at the time of the 1st dose. In a simple analysis, a consistent inverse relationship was observed between the quintile of VCLC and the risk of cholera in the placebo recipients and rings with radii up to 500 meters in Matlab. In contrast, we observed such relationship for rings with radii up to 200 meters in Kolkata. However, the results of the multivariable regression models showed association between VCLC of the OCV and the risk of cholera in placebo recipients with radii up to 150 meters in Kolkata, India and up to 500 meters in Matlab, Bangladesh (Table 1). Because vaccine herd protection only occurs for person-to-person transmission of cholera, these findings suggest that the geographic dimension of transmissions from one person to another person can be fairly large and may vary from one endemic area to another.

**Table 1:** The risk of cholera in placebo recipients to killed WC OCV coverage in different rings of populations in the Matlab, Bangladesh and Kolkata, India trials (16).

**Matlab, Bangladesh**				**Kolkata, India**			
**Ring size**	Odds ratio^*^	**95% CI**	***P*-value**	**Ring size**	Odds ratio^*^	**95% CI**	***P*-value**
0–100 m	0.98	0.97–0.99	0.03	0–50 m	0.98	0.97–0.99	0.03
101–200 m	0.98	0.97–0.99	0.01	51–100 m	0.97	0.95–0.99	0.01
201–300 m	0.98	0.97–0.99	0.01	101–150 m	0.97	0.95–0.99	0.02
301–400 m	0.98	0.97–0.99	0.02	151–200 m	0.99	0.96–1.01	0.36
401–500 m	0.98	0.97–0.99	0.03	201–250 m	1.00	0.97–1.03	0.85
501–600 m	1.00	0.98–1.01	0.87	251–300 m	0.98	0.95–1.01	0.14
601–700 m	1.00	0.98–1.01	0.43	301–350 m	1.00	0.97–1.03	0.82

* *Odds ratios adjusted for age at the date of first dose*.

## Measuring the Herd Protection by Killed WC OCV in an Individually Randomized Trial

**Figure 1:**
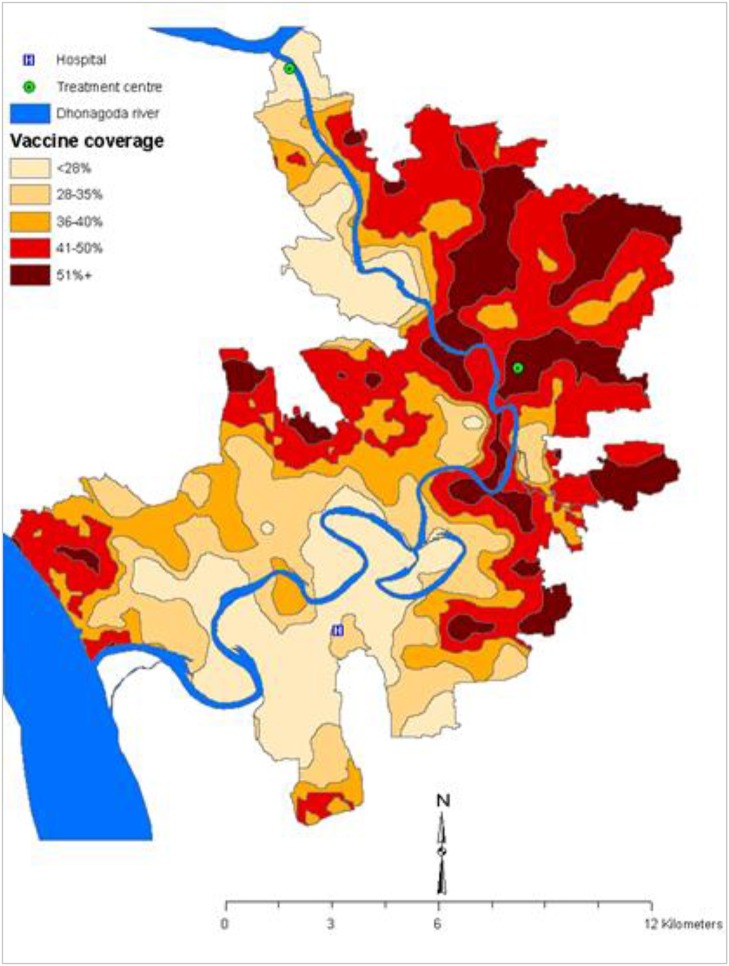
Spatial variation of the OCV coverage in the 1985 trial in Matlab, Bangladesh (3).

Until 2005, it was believed that the herd protection of a vaccine cannot not be measured in an individually randomized trial. Indeed such designs have been used intentionally to isolate the measurement of vaccine protection from vaccine herd effects, thus ensuring that only direct vaccine protection is measured. While this may be true in certain situations, our reanalysis of the individually randomized, placebo-controlled trial of killed WC OCVs in 2005 observed considerable spatial heterogeneity of killed WC OCV coverage in the trial population in a rural area of Bangladesh, attributed to spatial variations in refusal and ineligibility rate in the trial, so that some subpopulations had high enough levels of vaccine coverage for vaccine herd protection to come into effect (Figure 1). The heterogeneity in the killed WC OCV coverage in space allowed us to estimate vaccine herd protection with the logic that if the killed WC OCV conferred herd protection, then the risk of cholera among the analyzed individuals at the centers of virtual clusters would be inversely related to the killed WC OCV coverage in residents of virtual clusters surrounding these individuals, where the geographic boundaries of the virtual clusters were defined by GIS coordinates of residences. By analyzing the VCLC of killed WC OCV, computed as the number of persons who received two complete doses of a killed WC OCV, divided by the number of age- and gender- eligible individuals living within 500 m of the focal individual living in the center of the virtual cluster, the study evaluated the different measures of killed WC OCV herd protection of the population.

**Figure 2:**
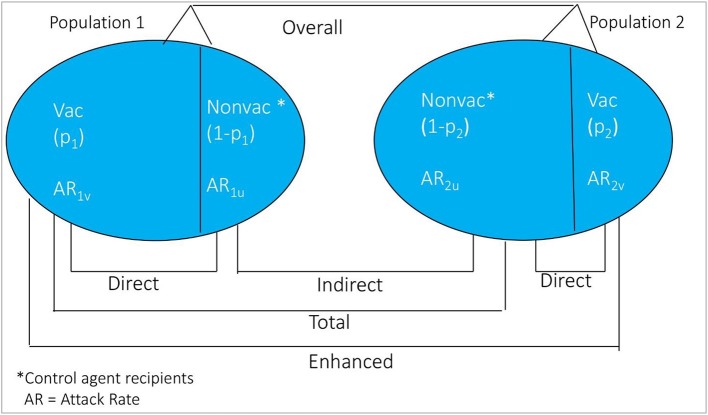
Conceptual diagram of the different measure of vaccine protection in individually randomized trial.

The conceptual diagram for measuring vaccine herd protection in an individually randomized trial is shown in Figure 2. Considered simplistically, the study population is classified into two groups: (i) population 1 consists of persons residing in virtual clusters whose VCLC of OCV is high, and (ii) population 2 comprises individuals residing in virtual clusters whose VCLC of OCV is low. Indirect vaccine protection can be calculated as one minus ratio of the cholera attack rates among non-vaccinated subjects between Population 1 and Population 2 [(1-AR_1u_/AR_2u_) × 100%]. Total vaccine protection can be calculated from rates of disease among vaccinated persons in Population 1 and among non-vaccinees in Population 2 [(1-AR_1v_/AR_2u_) × 100%]. Enhanced vaccine protection can be calculated as [(1-AR_1v_/AR_2v_) × 100%] and overall protection is calculated as 1-(AR in Population 1)/(AR in Population 2) x 100%. Note that direct protection can be calculated according to the standard formula for vaccine protective efficacy for each of the populations, as seen in the figure: [(1-AR_1v_/AR_1u_) × 100%] in Population 1 and [(1-AR_2v_/AR_2u_) x 100%] in Population 2.

By classifying the VCLC of killed WC OCV into quintiles of ascending vaccine coverage, we observed that the risk of cholera in persons who took at least two complete doses of placebo was inversely related to the VCLC of killed WC OCV as one moved from one level of coverage to the next higher level (indicating indirect vaccine protection); the same was true of the risk of cholera in vaccinees (indicating enhanced vaccine protection) (Table 2) (3). The trend of the relationship was more pronounced among placebo recipients than among vaccinees, as examined by Spearman's correlation coefficient. Correspondingly, the variation in the risk of cholera between the highest and lowest quintiles of VCLC of killed WC OCV was more marked for placebo recipients (1.47 vs. 7.01 cases per 1,000) than for vaccinees (1.27 vs. 2.66 cases per 1,000). The inverse relationship between the VCLC of killed WC OCV and the risk of cholera remained significant in a multivariable model that adjusted for the potential confounding variables. The relationship was significant when considering the risk of cholera in only placebo recipients (OR: 0.96; 95% CI: 0.94-0.98; *P* < 0.0001), and was of marginally significant in a model that considered the risk only in vaccinees (OR: 0.98; 95% CI: 0.96-1.00; *P* = 0.05). Estimates of vaccine protective efficacy, assessed conventionally as [1–(Relative risk of cholera in vaccinees vs. placebo recipients) X 100%] was higher when VCLC of killed WC OCV was 50% or less, but declined markedly when VCLC of killed WC OCV was over 50%, illustrating that higher VCLC of killed WC OCV in an individually randomized trial may reduce estimates of VE.

**Table 2:** Vaccine effectiveness (VE) among individuals who received OCV or placebo by VCLC of OCV during the first year of follow-up, Matlab, Bangladesh (3).

**Level of VCLC of OCV^‡^**	**Vaccinees**			**Placebo recipients**			**VE (%)^§^**	**95% CI for VE (%)**	***P*-value**
	***N***	**Cases**	Risk/1,000 persons^*^	***N***	**Cases**	**Risk/1,000 persons^†^**			
<28%	5,627	15	2.66	2,852	20	7.01	62	23 to 82	0.006
28–35%	8,883	22	2.47	4,429	26	5.87	58	23 to 77	0.003
36–40%	10,772	17	1.57	5,503	26	4.72	67	36 to 83	0.0004
41–50%	11,513	26	2.25	5,801	27	4.65	52	14 to 73	0.01
51%+	12,541	16	1.27	6,082	9	1.47	14	−111 to 64	0.89

* *Spearman's correlation coefficient: −0.90 (P = 0.08)*.

† *Spearman's correlation coefficient: −1.00 (P = 0.02)*.

‡ *Households are arranged by VCLC into quintiles, each with approximately same population size that was age- and gender-eligible to have participated in the trial*.

§ *Vaccine effectiveness for individuals residing in household with the cited VCLC*.

Although the trial was individually randomized, it was important to consider the fact that the virtual clusters themselves were not randomly allocated to high vs. low levels of vaccine coverage. To evaluate whether these inverse relationships could be non-specific reflections of a higher level of diarrhea in areas with lower turnover in the trial, the study evaluated whether there was an inverse correlation between the VCLC of killed WC OCV and the risk of a bias indicator condition, dysentery. Dysentery was analyzed as a bias indicator condition because it is a syndrome that is transmitted by the same fecal-oral route as cholera, has the similar clinical features, prompts patients to seek health care at similar provider sites, but is not caused by *Vibrio cholera* and is thus *not* expected to be prevented by killed WC OCV. Thus, analyses of vaccine protection against dysentery, conducted in the same fashion as for killed WC OCV, should fail to reveal vaccine herd protection if the analyses of herd protection against cholera had been unbiased (Table 3). Although the individuals living in a household in the lowest quintile of VCLC of killed WC OCV had the highest risk of dysentery (5.3 cases per 1,000), there was no relationship between the quintile of VCLC of killed WC OCV and the risk of dysentery, as determined by the Spearman's correlation coefficient test (correlation coefficient: −0.30, *P* = 0.62) suggesting that the estimates of herd protection by the killed WC OCV against cholera were not likely the result of an ecological bias.

**Table 3:** Risk of dysentery among recipients of OCV or placebo, by VCLC of OCV during the 1st year of follow-up, Matlab, Bangladesh (3).

**VCLC of OCV^†^**	**Target population**	**Recipients of 2 doses**	**Number of cases**	Risk per 1,000 persons^*^
<28%	24,954	8,479	45	5.30
28–35%	25,059	13,312	28	2.10
36–40%	24,583	16,275	49	3.01
41–50%	24,159	17,314	40	2.31
≥51%	22,394	18,623	48	2.58
Total	121,149	74,003	210	2.84

* *Spearman's correlation coefficient: −0.30 (P = 0.62)*.

† *Arranged into quintiles, each with approximately the same size of population that was age- and gender-eligible to have participated in the trial*.

In summary, this study illustrated for the first time that killed WC OCVs are capable of conferring herd as well as direct protection against cholera, and that appropriate analyses of individually randomized vaccine trials may yield important insights into vaccine herd protection.

## Measuring the Herd Protection by Killed WC OCV in a Cluster Randomized Trial

**Figure 3:**
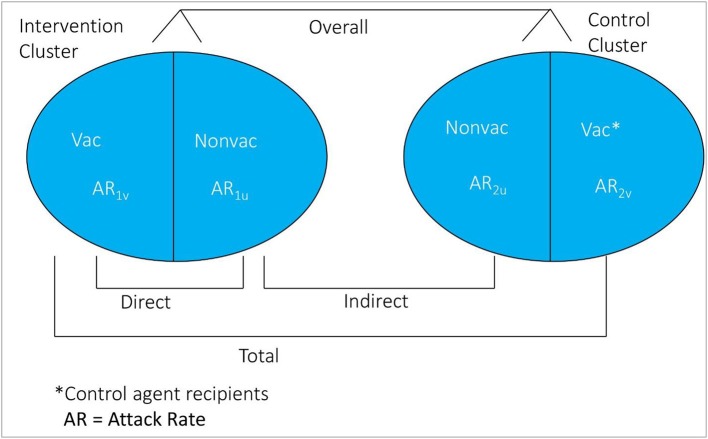
Conceptual diagram of the different measure of vaccine protection in cluster randomized trial.

If certain epidemiological assumptions can be fulfilled, the cluster-randomized trial design is considered the ideal design for evaluation of population level effectiveness of a vaccine (18). In this design, clusters of individuals are randomized to receive either of the study agents. Clusters are usually defined as geographic clusters, but may be defined by other features such as social networks. Rates of disease among recipients of assigned vaccine in the vaccinated clusters vs. the rate of disease among recipients of assigned control agent in the control clusters estimate total vaccine protection [(1-AR_1v_/AR_2v_) × 100%]. Rate among non-vaccinated persons in vaccinated clusters vs. rate among non-recipients of the control agent in the control clusters estimate indirect protection [(1-AR_1u_/AR_2u_) × 100%]. And, rates among all individuals in vaccinated clusters vs. all individuals in the control clusters estimate overall protection [1-(AR in Intervention Clusters)/(AR in the Control Clusters) × 100]. Direct vaccine protection is calculated in vaccinated clusters as [(1-AR_1v_/AR_1u_) × 100%] (Figure 3), though unlike estimates of indirect, total, enhanced, and overall vaccine protection, which are based on randomized comparisons of clusters, the estimates of direct vaccine protection are not based on randomized comparisons and are thus more susceptible to bias.

It is important to note that cluster randomized trials will measure vaccine herd protection only if the target infection is transmitted from person to person within the clusters, either direct or indirect way. Because measures of vaccine herd protection will be reduced if there is significant movement of the population into and out of the clusters, such trials should be undertaken in demographically stable populations. As well, use of this design to measure vaccine herd protection assumes that the cluster is the epidemiological unit of transmission of the target infection. If there is transmission of the infection between individuals in different clusters, or into the clusters from individuals outside the clusters, vaccine herd protection will be underestimated.

**Figure 4:**
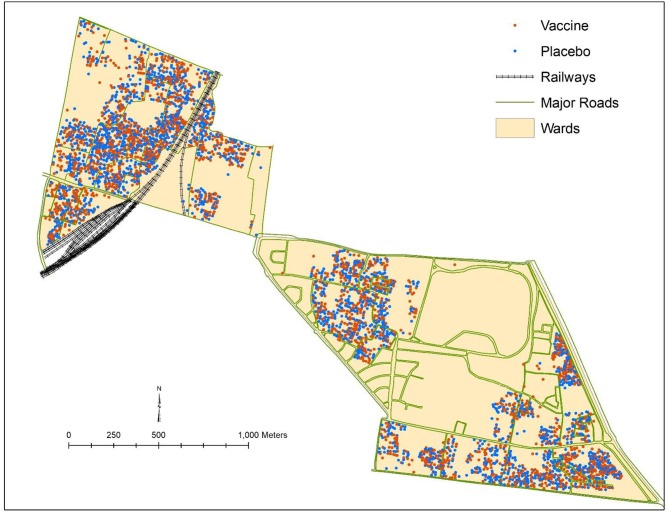
Vaccine and placebo clusters in the cluster randomized trial of OCV in Kolkata, India, 2006.

We conducted a cluster randomized trial of killed WC OCV in densely populated urban slums of Kolkata. Although residential dwellings were randomized to killed WC OCV or placebo, we found little evidence of vaccine herd protection when the clusters were assessed for total, indirect, and overall vaccine protection in conventional analyses (19). We suspected that the failure to detect herd protection by the killed WC OCV stemmed from a violation of the basic assumption of cluster randomized trials to measure vaccine herd protection that clusters be the epidemiological unit of transmission, with transmission of the target pathogen between members of the cluster but not to these members from outside the cluster (Figure 4). Clearly, in dense urban slums it would be unlikely that transmission of cholera occurs only within residential dwellings, the units of randomization for the trial. We therefore reanalyzed the trial using the VCLC approach that we had used for the individually randomized trial of killed WC OCV in Matlab, Bangladesh. With this approach, we demonstrated that a higher VCLC of killed WC OCV, computed using the population residing within a 250 meter radius of each analyzed individual, was linked with a lower risk of cholera for placebo recipients in households that had received placebo (19). Significant overall effectiveness was observed when VCLC of killed WC OCV was over 34% (Table 4). However, the herd protection of vaccinees in households allocated to killed WC OCV was not as pronounced as it was observed in the rural area of Bangladesh (3). The study nevertheless demonstrated that the risk of cholera among vaccinees and non-vaccinated individuals was significantly lower in areas with a high VCLC of killed WC OCV compared to those with low vaccination coverage area, confirming both total and indirect protection by the killed WC OCV.

**Table 4:** Vaccine effectiveness by CLVC of OCV among recipients and non-recipients of the OCV in the 3 year of follow-up, Kolkata, India (19).

**CLVC of OCV (%)**	**Indirect effectiveness (two doses of **placebo recipients**, ***n*****=34,968)****			**Overall effectiveness (All individuals including **non-participants in the trial**, ***n*****=108,349)****		
	VE (%)^**^	**95% C.I**.	***P*-value**	VE (%)^**^	**95% C.I**.	***P*-value**
0.00-25.00^*^	1	–	–	1.00	–	–
25.01–28.00	5	−58–43	0.85	−1	−48–32	0.95
28.01–31.00	50	6–74	0.03	32	−6–56	0.09
31.01–34.00	52	10–75	0.02	36	0–60	0.05
34.01+	65	22–85	0.01	54	28–74	0.01

* *Reference category*.

** *Calculated by 1-Hazard Ratio x 100 adjusted for the design effect of cluster randomization, age, individuals living in larger cluster, monthly per-capita expenditure of the household, living in their own house, living in a household always wash hands with soap & water after defecation, living in a household owning at least one luxury item, and distance from the household to the nearest health clinic*.

It might be queried why the conventional analysis of the clusters did not provide evidence of vaccine herd protection in this trial, but the approach using virtual clusters did. In the cluster design, the unit at risk for cholera was the group of individuals living in a dwelling unit, and the vaccine coverage of the residential dwelling was classified dichotomously as vaccinated or not according to the randomized assignment. The analysis was based on the assumption that there was little transmission of cholera into the cluster from the outside—an assumption, as noted earlier, that was very likely violated. The VCLC analytic approach differed in two important ways. First, vaccine coverage was not defined dichotomously as vaccinated or not, but on a dimensional scale reflecting the actual vaccine coverage in the population in the surrounding cluster. Second, in the VCLC approach, only the household at the center of the virtual cluster was analyzed for the risk of cholera. By making the virtual cluster sufficiently large in dimensions, this approach made it unlikely that the central household was infected by cholera from outside the clusters.

**Figure 5:**
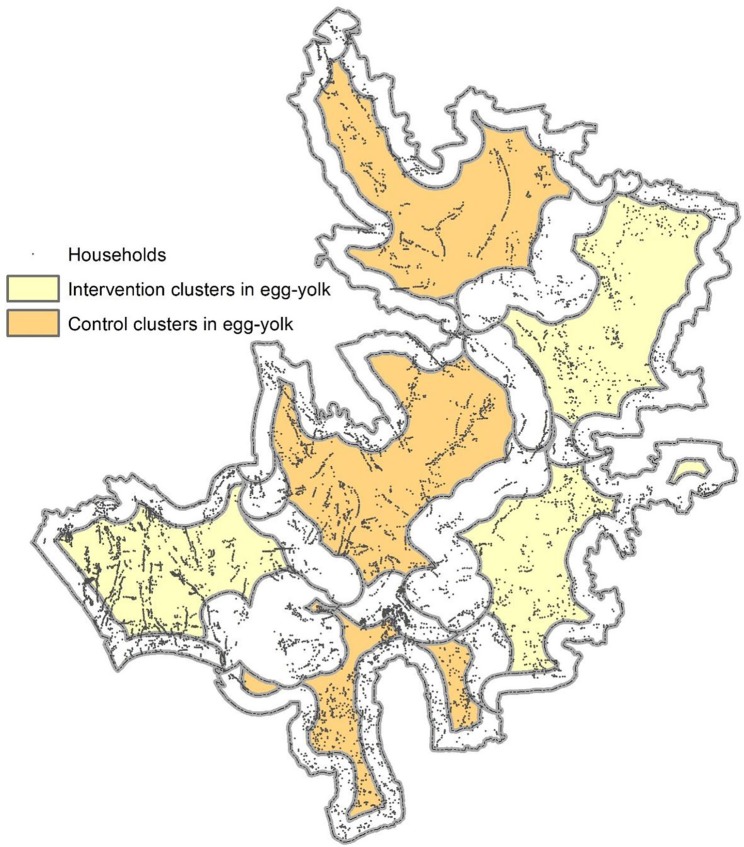
A sample map of the fried-egg design. Colored areas represent areas from where the household samples are to be selected for evaluation. The geographic features were obtained from the Matlab geographic information system database. The cluster boundaries were arbitrarily defined and have no connection with any studies conducted in Matlab. The areas in egg-white were made based on 500 meters from the perimeter of the arbitrarily defined clusters.

To minimize attenuation of measured levels of vaccine herd protection in cluster randomized vaccine trials, the fried-egg design has been proposed (20). In this design, clusters are randomized as before, but only individuals in the central part of each cluster (the “yolk” of the fried egg) are analyzed for the occurrence of the outcome (Figure 5). Details of the design and how we can evaluate the effectiveness of a vaccine under this design have been reported elsewhere (21).

## Measuring Herd Protection by Killed WC OCV in Observational Cohort Studies

We also measured herd protection by killed WC OCV in a setting where vaccines were given in a routine public health program, i.e., not in a randomized fashion. The analytical issues pertaining to measurement of vaccine herd protection in such observational cohort studies are similar to those for individually randomized trials. However, since there is no group randomized to a control agent in this design, indirect vaccine protection was estimated by evaluating the rate of cholera incidence among non-recipients of killed WC OCV in higher VCLCs of killed WC OCV vs. with those in lower VCLCs of killed WC OCV; total vaccine protection was estimated by evaluating the rate of cholera incidence in vaccinees in highest VCLCs of killed WC OCV vs. non-vaccinees in the lowest VCLCs of killed WC OCV; enhanced vaccine protection was estimated by evaluating the incidence of cholera in vaccinees in higher VCLCs of killed WC OCV vs. that in vaccinees in lower VCLCs of killed WC OCV; and overall vaccine protection was estimated by comparing the rate of cholera incidence among all individuals in higher VCLCs of killed WC OCV vs. all those in lower VCLCs of killed WC OCV. As in the analytical strategy for measuring vaccine herd protection in individually randomized trials, in which virtual clusters are not randomized to higher vs. lower levels of VCLC of killed WC OCV, these observational cohort analyses benefitted from analyses of bias indicator conditions, clinical outcomes that syndromically resembled cholera, was transmitted in the same fashion as cholera, and was cared for in the same manner and in the same settings as cholera, but was not expected to be prevented by the killed WC OCV under study. An absence of killed WC OCV herd protection in such analyses help verify that the primary analyses of killed WC OCV herd protection against cholera were not biased by residual confounding.

**Figure 6:**
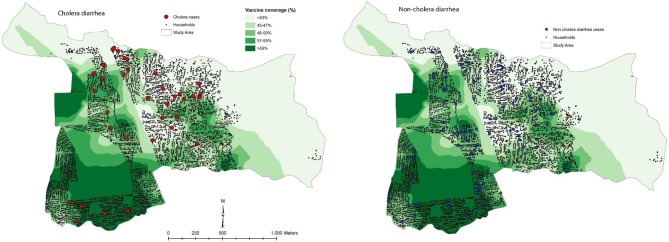
Spatial patterns of vaccine coverage and subsequent cholera and non-cholera diarrhea cases in the Unguja study site (22).

This approach was used in the evaluation of mass immunization with killed B subunit-WC OCV in our study in Zanzibar (22). In this study the investigators calculated the VCLC of killed WC OCV for residents in a 400 m radius around each household. Spatial patterns of the VCLC of killed WC OCV are presented in Figure 6, demonstrating considerable spatial heterogeneity in the VCLC of killed WC OCV in the study area. It is apparent from the map that most cholera cases were outside the high VCLCs of killed WC OCV. On the other hand, non-cholera cases appeared to be distributed randomly in space. We evaluated whether the rate of cholera incidence and non-cholera diarrhea (the latter being the bias indicator condition) in individuals who were unvaccinated decreased as VCLC of killed WC OCV increased. The risk of cholera by the VCLC of killed WC OCV in the analysis without any covariate showed a significant inverse relationship (*p* = 0.01) among the non-vaccine recipients (Table 5). Multivariable analyses that controlled for confounding variables showed a reduced relative risk of cholera with increasing coverage (0.95 for each percent increase in killed WC OCV coverage, 95% CI 0.90–0.99; *p* = 0.046), suggesting significant herd protection of the killed WC OCV. Such an inverse relationship was not observed for non-cholera diarrhea (*p* = 0.997). When the risk for cholera in non-vaccinated individuals was compared between the highest and lowest VCLCs of killed WC OCV, the risk of cholera, measuring indirect killed WC OCV protection, was reduced by 75% (95% CI: 11–93). Similarly, the study observed 80% total protection (95% CI: −71 to 98) among vaccinees in the highest VCLC of killed WC OCV compared with non-vaccinees in the lowest VCLC of killed WC OCV, a result that was underpowered by a small sample size. Overall protection among all residents in the study area was 72% (95% CI: 47–94) in the highest VCLC of killed WC OCV compared with the lowest VCLC of killed WC OCV. The study concluded that considerable herd protection by the tested killed WC OCV was observed in this sub-Saharan African setting.

**Table 5:** Risk for cholera and non-cholera diarrhea among different groups of people by VCLC of killed OCV around 400 m of the household during the 1 year of follow-up, Zanzibar, East Africa (16).

	**VCLC of OCV**	**Total subjects**	**Cholera**		**Non-cholera**	
			**Cases**	**Incidence rate/1000**	**cases**	**Incidence rate/1000**
No dose recipients	<39.38	4100	12	2.93	11	2.68
	39.38 – <43.78	4087	8	1.96	12	2.94
	43.78 – <46.85	4113	4	0.97	11	2.67
	46.85 – <53.95	4100	6	1.46	8	1.95
	53.95 –100	4100	3	0.73	13	3.17
	Total	20500	33	1.61	55	2.68
	Cochran-Amitage Trend Test (two-sided)		*p* –value = 0.0136		*p*-value = 0.997	
Two-dose recipients	<42.52	4781	5	1.05	42	8.78
	42.52 – <46.57	4787	0	0.00	39	8.15
	46.57 – <53.12	4781	0	0.00	22	4.60
	53.12 – <64.07	4775	0	0.00	15	3.14
	64.07 –100	4797	1	0.21	15	3.13
	Total	23921	6	0.25	133	5.56
	Cochran-Amitage Trend Test (two-sided)		*p*-value = 0.021		*p*-value <0.0001	
All eligible population	<40.93	9619	22	2.29	48	4.99
	40.93 – <44.85	9649	10	1.04	63	6.53
	44.85 – <50.08	9626	4	0.42	31	3.22
	50.08 – <62.09	9641	2	0.21	31	3.22
	62.09 –100	9643	4	0.41	34	3.53
	Total	48178	42	0.87	207	4.30
	Cochran-Amitage Trend Test (two-sided)		*p*-value <0.0001		*p*-value = 0.0030	

*Population is arranged by VCLC into quintiles, each having approximately the same size population in each level of VCLC*.

## Measuring Herd Protection by Killed WC OCV in Case-Control Studies

**Figure 7:**
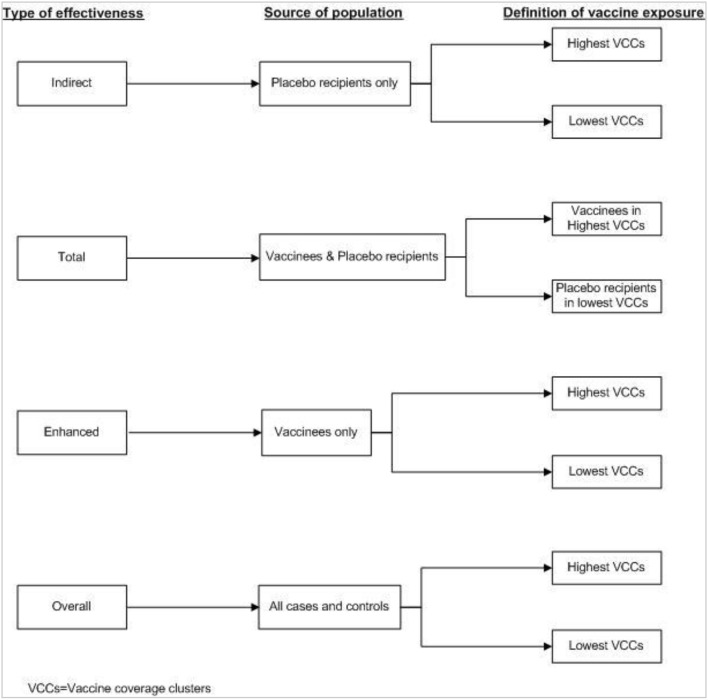
Definition of vaccine exposure by type of effectiveness in the case-control study (23).

The method for measuring population level effectiveness using a case-control study design is a bit complicated. In this approach, the cases are compared with controls (healthy subjects) selected from the same area. GIS is used to define virtual clusters and to calculate CLVC of killed WC OCV for each case and control. Figure 7 shows the source population and the definitions of vaccination exposures for the different types of killed WC OCV herd protection in case-control analyses. In this approach, “vaccine protection is measured by the odds ratios relating vaccine coverage of the cluster of residence (lowest vs. higher) to case-control status, where cases and controls are defined differently depending on the measure of protection. Indirect vaccine protection is measured among cases and controls who are not vaccinated; total protection is evaluated among cases and controls who are not vaccinated in the lowest vaccine coverage clusters or vaccinated in higher vaccine coverage clusters; overall protection is measured among all cases and controls; and enhanced protection is assessed among cases and controls who received the vaccine” (23).

We assessed the herd protection by killed WC OCV using a case-control study analytic design for the Kolkata trial described earlier (23). The VCLC of killed WC OCV was calculated using 250 m radius around in each case and control. Incidence density sampling was chosen for selecting cases and controls who lived in the study area at zero time (the time of vaccination). Cases were first episodes of cholera identified during 14 days after zero time and 1,095 days. For each case, four controls were randomly selected from the study area who did not have cholera until the date of onset of cholera of its matched case. Controls were matched to cases by age-group (<5 years, 5–14 years, and 15 years and older) at zero time. Vaccine effectiveness was computed as “1- odds ratio relating to case-control status in exposure categories) × 100%” (23).

To assess vaccine herd protection, we contrasted individuals whose virtual clusters were in the highest quintile of vaccine coverage (a VCLC of killed WC OCV of 34% or more) with persons who were in the lowest quintile of virtual cluster level coverage (VCLC of killed WC OCV of 25% or lower). We found that indirect vaccine protection was 76% (95% CI: 47–89%; *p* < 0.01), total vaccine protection was 75% (95% CI: 43–89%, *p* < 0.01, and overall vaccine protection was 44% (95% CI: -7–70%, *p* = 0.08) (Table 6).

**Table 6:** Different measures of killed WC OCV herd protection in case-control analyses (23).

**Measures of vaccine herd protection**	**No. of cases**	**No. of controls**	**Crude**			Adjusted^*^		
			**VE (%)**	**95% CI**	***p***	**VE (%)**	**95% CI**	***P***
Indirect	54	216	76	53–88	<0.0001	76	47-89	<0.01
Total	50	200	78	53–90	<0.0001	75	43-89	<0.01
Enhanced	16	64	−101	−558–39	0.25	−100	−558–39	0.25
Overall	95	380	52	15–72	0.0114	44	−7–70	0.08

* *Adjusted for age (at the time of 2nd dose for vaccine/placebo recipients and at the time of median date of 1st dose for placebo recipients and non-participants in the trial), living in a household owning at least one luxury item, distance from the household to the nearest health clinic. No covariates were adjusted for measuring enhanced effectiveness due to an insufficient number of cholera cases*.

There are several weaknesses of the case-control studies for assessing vaccine herd protection. Importantly, studies that do not consider the cluster-randomized design, in the case-control studies in which vaccine is given in an individually randomized fashion or according to routine practice, there are no clusters of control agent. Instead, this approach relies on use of low VCLC of killed WC OCV as a referent, possibly underestimating population level effectiveness. This design also requires a GIS for estimating of VCLC of killed WC OCV around the individuals. If these clusters are not based on a geographically referenced census database at the start of intervention, retrospective reconstruction of virtual clusters will be necessary, which may not always be feasible. Another issue shared by all approaches that require reconstruction of virtual clusters is the lack of site-specific information on what sizes and shapes constitute appropriate clusters. As mentioned earlier, we used a statistical criterion that was unrelated to the dimensions of cholera transmission for our analyses of herd protection by killed WC OCV. Ideally, one would have preferred use of a criterion that demarcated virtual clusters that are the epidemiological units of cholera transmission (19).

## Herd Protection by Killed WC OCV to Infants and Young Children

One potential drawback of killed WC OCV is its lower direct protection of children under 5 years (24). Another limitation is that these vaccines have not been evaluated and licensed for infants. Both drawbacks might be mitigated if vaccination of older persons in the population protected these young children against cholera by vaccine herd protection. The VCLC approach allowed us to reanalyze the Matlab field trial of killed OCVs in rural Bangladesh to evaluate whether vaccinating individuals 2 years and older with killed WC OCVs might protect young children (<2 years old) through vaccine herd protection, and if it is, whether these herd protection could be attributed to vaccination of a specific subpopulation, i.e., women 15 years or older or children 2-15 years old, who might play an important role in transmitting infection to these children <2 years old. The study showed that the risk of cholera in young children was inversely related to the CLVC of killed WC OCV in older children and adult women (the age-gender groups who were eligible to participate in the trial), ranging from 18.9 cases per 1,000 children residing in areas with the lowest quintile of killed WC OCV coverage to 8.6 cases per 1,000 children in areas with the highest quintile of killed WC OCV coverage (Table 7).

**Table 7:** Incidence of cholera among young children (<2 years)^*^ by CLVC of OCV during 1 year follow-up in the Bangladesh OCV trial, Matlab, Bangladesh (25).

**CLVC of OCV**	**Total No. of children (<24 months)**	**No. of cholera cases**	**Risk/1000^†^**
<28%	2,378	45	18.92
28–35%	2,371	27	11.38
36–40%	2,297	36	15.67
41–50%	2,207	29	13.14
51%+	2,205	19	8.61

* *Defined as children <24 months of age at the time of vaccination*.

† *P = 0.004 for trend*.

Since association between the risk of cholera and OCV coverage could have been driven by the demographic characteristics associated with the risk of cholera, the study fitted the data in a multivariable regression model in order for adjusting the potential demographic risk factors for cholera as observed in a study conducted in Matlab (26). However, the inverse relationship between VCLC of killed WC OCV and the risk of cholera in young children remained stable in the multivariable model, i.e., the relative risk of cholera was 0.98 for each percent increase in VCLC of killed WC OCV of older children and adult women (95% CI: 0.96–0.99; *P* < 0.001). The study was then evaluated whether the VCLC of killed WC OCV of a subgroup of the population was accountable for the observed herd effect in young children. The results yielded relative risk of cholera was 0.95 for each percentage increase in VCLC of killed WC OCV (95% CI: 0.92– 0.99; *P* < 0.01) of women over 15 years of age, but was 1.02 for each percentage increase in VCLC of killed WC OCV (95% CI: 0.98 −1.06; *p* = 0.24) of children 2-15 years, suggesting it was coverage of the adult women but not the coverage of children was important for protecting this young age group and that adult women likely were the sources of cholera transmitted to these young children.

## Conclusion

Mapping of study population with GIS played an important role in assessment of vaccine herd protection in every study design discussed in this article. The premise behind this GIS approach is that an individual interacts with others who resides in geographical proximity to him/her, forms an individual's contact network (27). “Such a network may help model the patterns of interactions among individuals that can promote transmission of an infectious disease” (28–30). The probability of becoming infected by a person, thus, depends on the number of infected individuals and vaccination coverage in that network. GIS allows us to define such network contact and to evaluate herd protection based on the vaccination coverage in that individual's network.

The main limitation in the GIS approach is that the virtual clusters may not be spatially random. For this reason, it was important to control for known demographic and other risk factors for cholera in multivariable models, which we used in the studies discussed earlier. In all of the studies, the inverse relationship between VCLC of killed WC OCV and the risk of cholera remained stable despite restrictions and analytic adjustments, making bias an implausible explanation for the observed associations.

The data on herd protection from our studies proved to be critical in the evaluation of the public health impact and cost-effectiveness of killed WC OCVs (31), and studies on herd protection may help policy decisions about vaccine introduction or continuation. It should be noted that the overall effectiveness of a vaccination program with killed WC OCV will depend on the population structure and coverage of the OCV among the different groups. In this situation, targeting the high-risk population will have a greater effect, as suggested by our analyses of protection of young children by vaccination of adult women, but not by vaccination of older children. Lastly, the duration of population level effectiveness of the killed WC OCV in a setting may not be directly proportional to the level of direct vaccination protection over time, thus emphasizing the need for analysis of both the level and duration of vaccine herd protection. Finally, it is worth noting that only the oral cholera vaccine studies have been included in this study, however, the findings on this study may be generalizable to other infectious diseases which have similar transmission mechanisms.

## Author Contributions

MA and JC contributed to the study design, data analysis, and wrote the manuscript.

### Conflict of Interest Statement

The authors declare that the research was conducted in the absence of any commercial or financial relationships that could be construed as a potential conflict of interest.

## References

[1] Anderson R May R. Vaccination and herd immunity to infectious diseases. Nature. (1985) 318:323–9. 10.1038/318323a0 3906406

[2] Longini IM Azhar N Ali M Yunus M Shenvi N Clemens JD Controlling endemic cholera with oral vaccine. PLoS Med. (2007) 4:e336 10.1371/journal.pmed.0040336 18044983 PMC2082648

[3] Ali M Emch M von Seidlein L Yunus M Sack DA Rao M. Herd immunity conferred by killed oral cholera vaccines in Bangladesh: a reanalysis. Lancet. (2005) 366:44–9. 10.1016/S0140-6736(05)66550-6 15993232

[4] Clemens JD Sack DA Harris JR Van Loon F Chakraborty J Ahmed F. Field trial of oral cholera vaccines in Bangladesh: results from three-year follow-up. Lancet. (1990) 335:270–3. 10.1016/0140-6736(90)90080-O 1967730

[5] Martin S Costa A Perea W. Stockpiling oral cholera vaccine. Bull World Health Organ. (2012) 90:714. 10.2471/BLT.12.112433 23109735 PMC3471062

[6] Longini IM Jr Halloran ME Nizam A. Model-based estimation of vaccine effects from community vaccine trials. Statist Med. (2002) 21:481–95. 10.1002/sim.994 11836731

[7] Ross R An application of the theory of probabilities to the study of a priori pathometry, part 1. Proc R Soc Lond Ser A. (1916) 92:204–30. 10.1098/rspa.1916.0007

[8] Struchiner CJ Halloran ME Robins JM Spielman A. The behaviour of common measures of association used to assess a vaccination programme under complex disease transmission patterns–a computer simulation study of malaria vaccines. Int J Epidemiol. (1990) 19:187–96. 10.1093/ije/19.1.187 2351514

[9] Halloran ME Longini IM Jr Struchiner CJ. Design and interpretation of vaccine field studies. Epidemiol Rev. (1999) 21:73–88. 10.1093/oxfordjournals.epirev.a017990 10520474

[10] Anderson RM May RM. Immunisation and herd immunity. Lancet. (1990) 335:641–5. 10.1016/0140-6736(90)90420-A 1969023

[11] Fine PE. Herd immunity: history, theory, practice. Epidemiol Rev. (1993) 15:265–302. 10.1093/oxfordjournals.epirev.a036121 8174658

[12] Fox J Elveback L Scott W Gatewood L Ackerman E. Herd immunity: basic concept and relevance to public health immunization practices. Am J Epidemiol. (1995) 141:187–97. 10.1093/oxfordjournals.aje.a117420 7840092

[13] Greenland S Frerichs RR. On measures and models for the effectiveness of vaccines and vaccination programmes. Int J Epidemiol. (1988) 17:456–63. 10.1093/ije/17.2.456 3403141

[14] Robbins JB Schneerson R Anderson P Smith DH. The 1996 Albert Lasker Medical Research Awards. Prevention of systemic infections, especially meningitis, caused by Haemophilus influenzae type b Impact on public health and implications for other polysaccharide-based vaccines. JAMA. (1996) 276:1181–5. 10.1001/jama.276.14.1181 8827975

[15] Halloran ME Struchiner CJ. Study designs for dependent happenings. Epidemiology. (1991) 2:331–8. 10.1097/00001648-199109000-00004 1742381

[16] Ali M Kim DR Kanungo S Sur D Manna B Digilio L. Use of oral cholera vaccine as a vaccine probe to define person-to-person transmission of cholera. Int J Infect Dis. (2018) 66:90–5. 10.1016/j.ijid.2017.11.020 29174695 PMC7413038

[17] Ali M Park JK Thiem VD Canh DG Emch M Clemens JD. Neighborhood size and local geographic variation of health and social determinants. Int J Health Geograph. (2005) 4:12. 10.1186/1476-072X-4-12 15927082 PMC1156930

[18] Donner A Klar N Design and Analysis of Cluster Randomization Trials in Health Research. London: Arnold (2000). 10.1191/096228000669355658

[19] Ali M Sur D You YA Kanungo S Sah B Manna B. Herd protection by a bivalent-killed-whole-cell oral cholera vaccine in the slums of Kolkata, India. Clin Infect Dis. (2013) 56:1123–31. 10.1093/cid/cit009 23362293

[20] Hayes RJ Moulton LH Cluster Randomized Trials. 2nd Edn. Boca Raton, FL; London; New York, NY: CRC Press; Taylor & Francis Group (2017).

[21] Ali M Qadri F Kim DR Islam T Im J Ahmmed F. Unmasking herd protection by an oral cholera vaccine in a cluster-randomized trial. Int J Epidemiol. (2019). 10.1093/ije/dyz060 [Epub ahead of print]. 30968110 PMC6693801

[22] Khatib AM Ali M von Seidlein L Kim DR Hashim R Reyburn R. Effectiveness of an oral cholera vaccine in Zanzibar: findings from a mass vaccination campaign and observational cohort study. Lancet Infect Dis. (2012) 12:837–44. 10.1016/S1473-3099(12)70196-2 22954655

[23] Ali M You YA Kanungo S Manna B Deen JL Lopez AL. Assessing different measures of population-level vaccine protection using a case-control study. Vaccine. (2015) 33:6878–83. 10.1016/j.vaccine.2015.07.045 26364121

[24] Bi Q Ferreras E Pezzoli L Legros D Ivers LC Date K. Protection against cholera from killed whole-cell oral cholera vaccines: a systematic review and meta-analysis. Lancet Infect Dis. (2017) 17:1080–8. 10.1016/S1473-3099(17)30359-6 28729167 PMC5639147

[25] Ali M Emch M Yunus M Sack D Lopez AL Holmgren J Vaccine protection of Bangladeshi infants and young children against cholera: implication for vaccine deployment and person-to-person transmission. Pediatr Infect Dis. (2008) 27:33–7. 10.1097/INF.0b013e318149dffd 18162935

[26] Glass RI Becker S Huq MI Stoll BJ Khan MU Merson MH. Endemic cholera in rural Bangladesh, 1966–1980. Am J Epidemiol. (1982) 116:959–70. 10.1093/oxfordjournals.aje.a113498 7148820

[27] McPherson M Smith-Lovin L Cook J Birds of a feather: Homophily in social networks. Ann Rev Sociol. (2001) 27:415–44. 10.1146/annurev.soc.27.1.415

[28] Meyers L Newman M Martin M Schrag S Applying network theory to epidemics: control measures for Mycoplasma pneumoniae outbreaks. Emerg Infect Dis. (2003) 9:204–10. 10.3201/eid0902.020188 12603991 PMC3369603

[29] Morris M Network Epidemiology: A Handbook for Survey Design and Data Collection. Oxford: Oxford University Press (2005). p. 252.

[30] Meyers L Contact network epidemiology: bond percolation applied to infectious disease prediction and control. Bull Am Math Soc. (2007) 44:63–86. 10.1090/S0273-0979-06-01148-7

[31] Clemens J Shin S Ali M New approaches to the assessment of vaccine herd protection in trials conducted before licensure. Lancet Infect Dis. (2011) 11:482–7. 10.1016/S1473-3099(10)70318-2 21616458

